# Cardiovascular Health Score and the Risk of Cardiovascular Diseases

**DOI:** 10.1371/journal.pone.0131537

**Published:** 2015-07-08

**Authors:** Congliang Miao, Minghui Bao, Aijun Xing, Shuohua Chen, Yuntao Wu, Jun Cai, Youren Chen, Xinchun Yang

**Affiliations:** 1 Department of Cardiology, Second Affiliated Hospital of Shantou University Medical College, Shantou, Guangdong, China; 2 Department of Cardiology, Chaoyang Hospital, Capital Medical University, Beijing, China; 3 Department of Cardiology, Kailuan Hospital, Hebei United University, Tangshan, China; University of Louisville, UNITED STATES

## Abstract

In 2010 the American Heart Association proposed a definition of ideal health behaviors and health factors to measure cardiovascular health, from which Huffman et al. created the Cardiovascular Health Score (CVH score) to estimate these metrics on an individual level. We performed a prospective cohort study among employees of the Kailuan Group Corporation, who underwent a physical examination in 2006–2007 to investigate the relationship between the CVH score and the risk of cardiovascular disease (CVD). A total of 91,598 individuals free of stroke and myocardial infarction at baseline were included in the final analysis. We calculated baseline CVH score for each metric (poor=0, intermediate=1, ideal=2 points; range=0–14 points for all seven metrics) and categorized them into three groups: inadequate (0–4 points), average (5–9 points), and optimum (10–14 points). Incidence of total number of CVD events, myocardial infarction, and stroke was analyzed among these three groups and each incremental point on the CVH score. During an average 6.81 years of follow-up, there were 3276 CVD events, 2579 strokes and 747 myocardial infarction occurred. After adjusting for several confounding factors, each better health category of the CVH score was associated with reduced odds of 47% for all CVD events, and each point higher on the CVH score was associated with reduced odds of 18%. Similar trends were detected in the risks for myocardial infarction and stroke. A higher CVH score is therefore a protective factor for CVD, myocardial infarction, and stroke.

## Introduction

Cardiovascular disease (CVD) is the leading cause of death and disability worldwide, and is a huge drain on public health expenditure [1,2]. To achieve the goal of reducing CVD mortality by 20% and improving cardiovascular health by 20% by the year 2020, in 2010 the American Heart Association (AHA) proposed a definition of ideal cardiovascular health behaviors and health factors [3]. This model contains seven metrics—four health behaviors (smoking, diet, physical activity, and body weight) and three health factors (plasma glucose, cholesterol, and blood pressure)—which are used to categorize the population as being in “poor,” “intermediate,” and “ideal” cardiovascular health. To estimate cardiovascular health status on an individual level, Huffman et al. created the AHA Cardiovascular Health Score (CVH score), which includes all seven cardiovascular health behaviors and factors (poor, 0 points; intermediate, 1 point; ideal, 2 points; for a total score ranging from 0 to 14 points) [4].

Several previous studies have indicated that with increasing numbers of ideal cardiovascular health metrics, the incidence of CVD, stroke, myocardial infarction, and all-cause mortality has decreased gradually [5–10]. A recent study found that with increasing CVH score, the incidence of stroke decreased [11]. However, individuals with the same numbers of ideal metrics probably have other metrics in different categories. In fact, in comparison with persons with more “poor” metrics, those with more “intermediate” metrics tend to have divergent levels of cardiovascular health. Thus, merely estimating the number of ideal metrics might fail to account for intermediate and poor status. By comparison, the CVH score gives fuller consideration to the intermediate and poor categories and estimates more meticulously the health status of individuals. However, to date, the relationship between CVH score and the risk of CVD has not been reported. Therefore, we investigated the relationship between CVH score and the risk of CVD events, myocardial infarction, and stroke in the population of the Kailuan study (Clinical Trial Registration identifier ChiCTR-TNC-11001489).

## Subjects and Methods

Written informed consent was obtained from all participants. The Ethics Committee of the Kailuan General Hospital and Beijing Chaoyang Hospital approved the study.

### Study Participants

The Kailuan study is a prospective cohort study focusing on the Kailuan community in Tangshan, a large modern city in northern China, where 11 hospitals are responsible for the health care of the community, all of which participated in conducting physical examinations. A total of four physical examinations were performed during 2006–2007, 2008–2009, 2010–2011, and 2012–2013, respectively, and 101,510 workers participated in these examinations (81,110 men and 20,400 women), including both in-service and retired workers.

### Inclusion and Exclusion Criteria

Subjects were included if they were ≥18 years old, participated in physical examination during 2006–2007, and provided informed consent for the present study. Exclusion criteria were as follows: history of stroke, history of myocardial infarction, incomplete information from the seven cardiovascular health metrics and women were pregnant and lactating at the time when measurements were conducted (described below).

### Data Collection

#### General data collection and anthropometric measurements

Methods of epidemiological questionnaire filling and anthropometric measurements were carried out according to the standard protocols described in published literature by our group [8,12]. Questionnaires were collected in person by trained doctors. Information included demographic and socioeconomic data, medical history, family medical history, alcohol consumption, smoking status, dietary data, physical activity, education level, and average income. The anthropometric measurements included height, weight, waist circumference, hip circumference, and body mass index (BMI). All of the relevant measurements were performed by trained researchers in strict accordance with the standards of measurement. A corrected RGZ-120 scale was used to measure height and weight. Individuals to be measured were required to be thinly clothed, with shoes and hats removed. Height measurements were accurate to 0.1 cm and weight to 0.1 kg. BMI was calculated as body weight divided by height squared (kg/m^2^). Heart rate was recorded by 12-lead electrocardiogram (ECG). The RR interval was regarded as resting heart rate.

#### Blood pressure measurement

Blood pressure (BP) was measured on the left arm using a mercury sphygmomanometer with a cuff of appropriate size following the standard recommended procedures. Individuals were required to stop smoking and stop drinking tea or coffee for more than 30 min, then to sit and rest for 15 min. Three BP readings were taken at 5-min intervals, with the average used for data analysis. If the three measurements differed from each other by >5 mmHg, additional readings were performed, and the average of these readings was regarded as the final BP value.

#### Biochemical measurements

Measurements included fasting blood glucose (FBG), triglyceride (TG), total cholesterol (TC), high-density lipoprotein cholesterol, low-density lipoprotein cholesterol, uric acid, and high-sensitivity C-reactive protein (CRP). Blood samples (5 mL) from the antecubital vein were collected between 7:00 am and 9:00 am after an overnight fast. All biochemical variables were measured using an automatic analyzer (Hitachi 7600).

#### Follow-up and identification of events

CVD events included myocardial infarction and stroke. Each participant could contribute only one end point. If two or more events occurred, the first one was regarded as the end point of observation. Participants were followed up every 2 years. The follow-up began with the physical examination between June 2006 and October 2007 and continued through December 31, 2013 or until the occurrence of cardiovascular events (i.e., myocardial infarction and stroke), the date of death, or loss to follow-up. Information on outcomes was further confirmed by checking discharge summaries from the 11 hospitals and medical records from medical insurance companies. For those participants without face-to-face follow-up, the outcomes information was obtained directly by checking death certificates from provincial vital statistics offices, discharge summaries, and medical records. All events were identified and recorded by trained researchers and doctors every 6 months.

#### Diagnostic criteria

Diagnostic criteria for myocardial infarction were based on chest pain symptoms, ECG changes, and myocardial necrosis biomarkers. Stroke was categorized into three main types: brain infarction, cerebral hemorrhage, and subarachnoid hemorrhage. Diagnostic criteria for stroke were based on computed tomography or magnetic resonance imaging [13].

### Definitions of Cardiovascular Health Metrics

According to the cardiovascular health behaviors and health factors proposed by the AHA [3] and the CVH score devised by Huffman et al [4]. seven behaviors and factors were classified into three levels (poor = 0 points; intermediate = 1 point; ideal = 2 points; total score: 0–14 points). Participants were further categorized into three cardiovascular health groups: inadequate (0–4 points), average (5–9 points), and optimum (10–14 points). Detailed definitions of “poor,” “intermediate,” and “ideal” levels for all seven metrics are given below.

#### Health behaviors

Health behaviors were categorized as follows. Cigarette smoking: ideal, never smoker; intermediate, used to smoke but not now; poor, current smoker. BMI: ideal, <25 kg/m^2^; intermediate, 25–30 kg/m^2^; poor, ≥30 kg/m^2^. Physical activity: ideal, ≥80 min/week; intermediate, 0–80 min/week; poor, never exercise. Since salt intake greatly affects cardiovascular health in the Chinese population, our questionnaire included salt intake rather than vegetable intake as a health behavior [14]. Diet categories were as follows: ideal, light salt intake; intermediate, moderate salt intake; poor, heavy salt intake.

#### Health factors

Untreated TC <200 mg/dL was considered ideal, untreated TC 200–239 mg/dL or treated TC <200 mg/dL intermediate, and TC ≥240 mg/dL poor. Untreated systolic BP (SBP) <120 mmHg (1 mmHg = 0.133 kPa) and diastolic BP (DBP) <80 mmHg was considered ideal, SBP 120–139 mmHg or DBP 80–89 mmHg or treated BP <120/80 mmHg intermediate, and SBP ≥140 mmHg or DBP ≥90 mmHg poor. Untreated FBG <100 mg/dL was considered ideal, FBG 100–125 mg/dL or treated FBG <100 mg/dL intermediate, and FBG ≥126 mg/dL poor.

### Statistical Methods

SPSS 13.0 software (SPSS, Chicago, IL, USA) was used for statistical analysis. Normally distributed continuous variables were recorded as mean ± SD, and skewness distributed data were converted by logarithmic transformation. Continuous variables were compared using analysis of variance. Categorical variables were described as percentages and compared by the chi-square test. The incidence density of cardiovascular events was recorded as per 1,000 person-years. Cox proportional hazards models were used to analyze the hazard ratio and 95% confidence interval (CI) of the incidence of total CVD events, myocardial infarction, and stroke in different categories (inadequate, average, and optimum) and per point incrementally on the CVH score. Mode 1 was a single-factor analysis model; mode 2 was adjusted for age, gender, alcohol consumption, income, education and history of cardiovascular disease on the basis of mode 1; and mode 3 was further adjusted for heart rate, blood uric acid, and high-sensitivity CRP on the basis of mode 2. A value of *P*<0.05 (bilateral) was regarded as statistically significant. In addition, to estimate each cardiovascular health metric when accounting for the associations between CVH score and CVD incidence, we omitted each metric one at a time and re-analyzed these associations.

## Results

**Fig 1:**
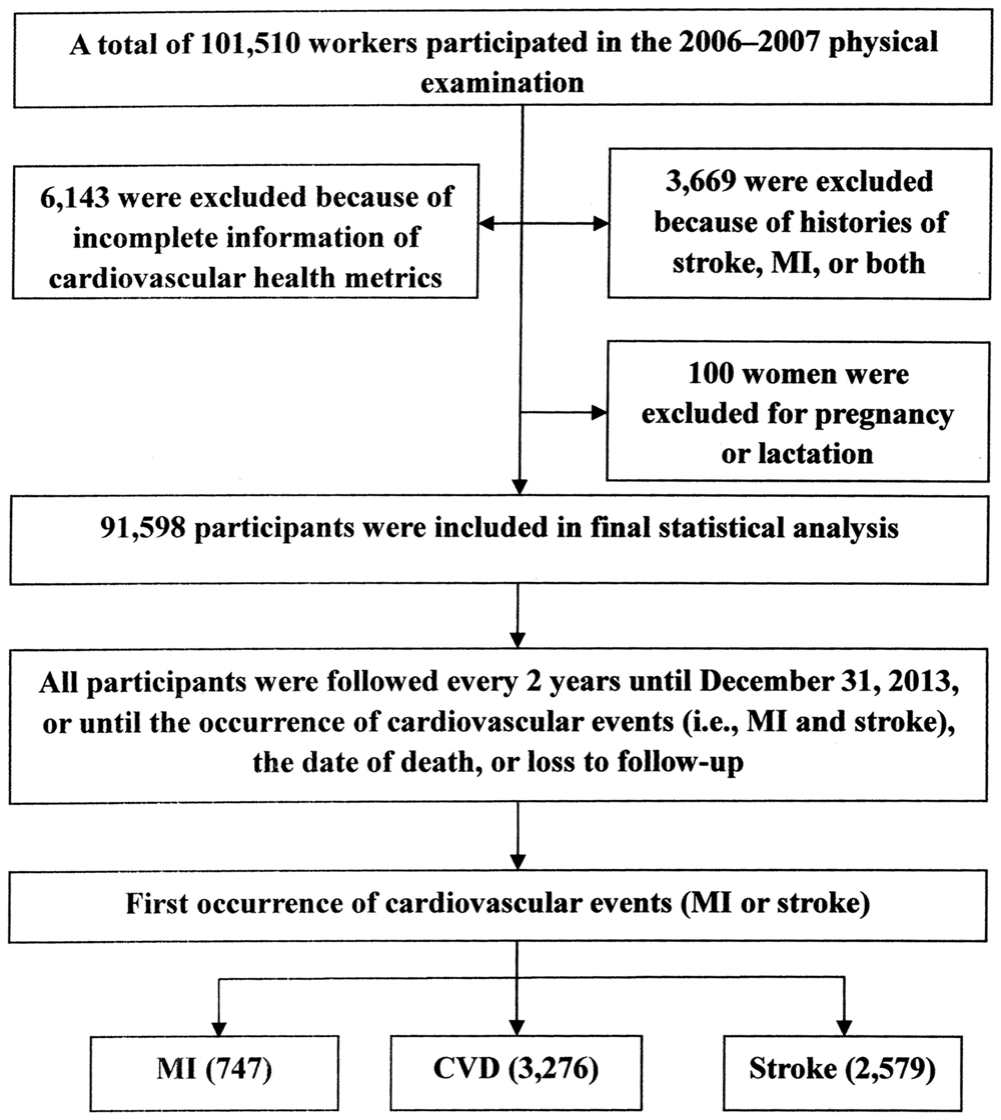
Flow Chart of the Study. Stroke was categorized into three main types: brain infarction, cerebral hemorrhage, and subarachnoid hemorrhage. CVD, cardiovascular disease; MI, myocardial infarction.

A total of 101,510 workers participated in the 2006–2007 physical examination, from whom 9,812 were excluded: 1,113 with history of stroke, 2,353 with history of myocardial infarction, 203 with history of both stroke and myocardial infarction, 6,143 lacking information regarding the seven metrics and 100 women were pregnant and lactating at the time when measurements were conducted. A total of 91,598 participants (90.24%) were included in the observation cohort. Details are shown in Fig 1.

### Baseline Characteristics of Different Groups

The baseline characteristics of participants are shown in Table 1. Of 91,598 participants, 72,826 were male and 18,772 female. The average age was 51.55 ± 12.36years (range 18–98), and the average CVH score was 8.64 ± 2.00 points. The number of participants categorized into the three cardiovascular health groups according to CVH score was 2,355 in the inadequate group (0–4 points, 2.57% of 91,598 participants), 56,720 in the average group (5–9 points, 61.92%), and 32,523 in the optimum group (10–14 points, 35.51%). A better health category of CVH score was associated with a higher proportion of young people, higher education level, and higher proportion of nondrinkers (*P*<0.05). Conversely, male proportion, heart rate, SBP, DBP, BMI, FBG, TC, high-sensitivity CRP, uric acid, hypertension, diabetes, history of cardiovascular diease and lower income were negatively associated with a better health category of CVH score (*P*<0.05).

**Table 1:** Baseline Characteristics of Different Groups According to the CVH Score at Baseline.

Characteristic	Total participants	Inadequate	Average	Optimum	*P*
	*n* = 91598	*n* = 2355	*n* = 56720	*n* = 32523	
Men, (*n*%)	72826 (79.5)	2284 (97.0)	49307 (86.9)	21235 (65.3)	<0.01
Age, years	51.6±12.4	51.2±9.7	52.6±11.7	49.7±13.4	<0.01
Age, years (%)					<0.01
<40	14748 (16.1)	249 (10.6)	7148 (12.6)	7351 (22.6)	
40–59	55530 (60.6)	1709 (72.6)	35489 (62.6)	18332 (56.4)	
≥60	21320 (23.3)	397 (16.9)	14083 (24.8)	6840 (21.0)	
Heart Rate, /min	73.9±10.1	77.8±10.9	74.5±10.2	72.4±9.7	<0.01
SBP, mmHg	130.6±20.9	147.4±20.0	136.0±20.3	119.8±17.1	<0.01
DBP, mmHg	83.5±11.8	94.0±11.6	86.5±11.4	77.4±9.7	<0.01
BMI, kg/m^2^	25.0±3.5	28.6±3.5	25.9±3.4	23.3±2.7	<0.01
FBG, mg/dL	99.1±30.2	135.0±49.2	103.5±33.6	88.9±13.8	<0.01
TC, mg/dL	191.5±44.8	240.8±47.7	198.5±46.6	175.7±34.4	<0.01
UA, μmol/L	289.0±83.0	324.4±102.0	298.7±83.9	269.5±75.7	<0.01
hsCRP	-0.12±0.66	0.05±0.61	-0.08±0.64	-0.21±0.68	<0.01
Hypertension (%)	39302 (42.9)	2051 (87.1)	31540 (55.6)	5711 (17.6)	<0.01
Diabetes (%)	8160 (8.9)	1054 (44.8)	6754 (11.9)	352 (1.1)	<0.01
Education (%)					<0.01
Illiteracy/primary	9287(10.1)	389 (16.6)	6556 (11.6)	2342 (7.2)	
Middle school	75891 (82.9)	1848 (78.7)	47169 (83.2)	26874 (82.7)	
College/University	6360 (6.9)	112 (4.8)	2955 (5.2)	3293 (10.1)	
Income, ¥ (%)					<0.01
<600^a^	26181 (28.6)	1100 (46.8)	17936 (31.7)	7145 (22.0)	
600–1000^a^	59275 (64.7)	1071 (45.6)	35189 (62.1)	23015 (70.8)	
≥1000^a^	6067 (6.6)	179 (7.6)	3542 (6.3)	2346 (7.2)	
Alcohol (%)					<0.01
Never	54130 (59.1)	519 (22.1)	29108 (51.4)	24503 (75.4)	
Ex-user	3132 (3.4)	144 (6.1)	2287 (4.0)	701 (2.2)	
User	34286 (37.4)	1691 (71.8)	25285 (44.6)	7310 (22.5)	
History of cardiovascular disease	5425 (5.9)	247 (10.5)	3535 (6.2)	1643 (5.1)	

SBP, systolic blood pressure; DBP, diastolic blood pressure; BMI, body mass index; FBG, fasting blood glucose; TC, total cholesterol; UA, uric acid; hsCRP, high-sensitivity C-reactive protein.

^a^Average monthly income of every family member.

### Incidence of Cardiovascular Events in Different Groups

The incidence of cardiovascular events in different groups is shown in Table 2. A total of 3,276 CVD events occurred during an average of 6.81 years of follow-up. The incidence of cardiovascular events in the three groups was 10.47, 6.39, and 2.93 per 1000 person-years. A Cox proportional hazards model was performed with cardiovascular events as dependent variable and the three CVH groups as independent variable (with the inadequate group as reference). Using model 1, compared with the inadequate group the hazard ratio (95% CI) of cardiovascular events for the average and optimum groups were 0.61 (0.52–0.71) and 0.28 (0.24–0.33), respectively. Using model 2, adjusted for age, gender, alcohol consumption, income, education and history of cardiovascular disease on the basis of model 1, the corresponding values were 0.52 (0.45–0.62) and 0.26 (0.22–0.32); and using model 3, further adjusted for heart rate, blood uric acid, and high-sensitivity CRP on the basis of model 2, these values were 0.56 (0.48–0.66) and 0.29 (0.24–0.35). Each better health category of CVH score was associated with reduced odds of 47%. Each point higher on the CVH score was associated with reduced odds of 18%. These results indicated that a higher CVH score was negatively associated with incidence of CVD events (*P* for trend <0.01).

**Table 2:** Incidence of Total CVD Events, Myocardial Infarction, and Stroke among Three Groups According to the CVH Score at Baseline.

Characteristic	CVH score		
	Inadequate (0–4)	Average (5–9)	Optimum (10–14)
	*n* = 2355	*n* = 56720	*n* = 32523
Total CVD events			
Cases	165	2458	653
Person-years	15765.1	384844.5	222988.4
Incidence rate	10.47	6.38	2.93
Stroke			
Cases	129	1937	513
Person-years	15901.4	386385.6	223403.8
Incidence rate	8.11	5.01	2.30
Myocardial infarction			
Cases	39	563	145
Person-years	16142.8	390842.6	224514.2
Incidence rate	2.42	1.44	0.65

Incidence rate is per 1000 person-years.

### Incidence of Stroke in Different Groups

The incidence of stroke in different groups is shown in Table 3. A total of 2,579 strokes occurred during follow-up. The incidence of stroke in the three groups was 8.11, 5.01, and 2.30 per 1000 person-years, respectively (Table 2). The hazard ratios (95% CI) of stroke for the average and optimum groups, respectively, in comparison with the inadequate group were: model 1, 0.62 (0.52–0.74) and 0.28 (0.23–0.34); model 2, 0.54 (0.45–0.65) and 0.28 (0.23–0.34); model 3, 0.57 (0.48–0.69) and 0.30 (0.24–0.37). Each better health category of CVH score was associated with reduced odds of 46%, and each point higher on the CVH score was associated with reduced odds of 18%. The results indicated that a higher CVH score correlated negatively with incidence of stroke (*P* for trend <0.01).

**Table 3:** Hazard Ratios (95% CI) of Incidence of Total CVD Events, Myocardial Infarction, and Stroke among Different Groups According to the CVH Score at Baseline.

Characteristic	Cases, *n*	Model 1		Model 2		Model 3	
		HR (95% CI)	*P*	HR (95% CI)	*P*	HR (95% CI)	*P*
CVD		0.49 (0.46–0.53)	<0.01	0.51 (0.47–0.55)	<0.01	0.53 (0.49–0.57)	<0.01
Inadequate	165	1		1		1	
Average	2458	0.61 (0.52–0.71)	<0.01	0.52 (0.45–0.62)	<0.01	0.56 (0.48–0.66)	<0.01
Optimum	653	0.28 (0.24–0.33)	<0.01	0.26 (0.22–0.32)	<0.01	0.29 (0.24–0.35)	<0.01
*P* for trend		<0.01		<0.01		<0.01	
Each point higher CVH score		0.82 (0.80–0.83)	<0.01	0.81 (0.79–0.82)	<0.01	0.82 (0.80–0.84)	<0.01
Stroke		0.50 (0.46–0.54)	<0.01	0.52 (0.48–0.56)	<0.01	0.54 (0.49–0.58)	<0.01
Inadequate	129	1		1		1	
Average	1937	0.62 (0.52–0.74)	<0.01	0.54 (0.45–0.65)	<0.01	0.57 (0.48–0.69)	<0.01
Optimum	513	0.28 (0.23–0.34)	<0.01	0.28 (0.23–0.34)	<0.01	0.30 (0.24–0.37)	<0.01
*P* for trend		<0.01		<0.01		<0.01	
Each point higher CVH score		0.82 (0.81–0.84)	<0.01	0.81 (0.80–0.83)	<0.01	0.82 (0.81–0.84)	<0.01
Myocardial infarction		0.48 (0.42–0.56)	<0.01	0.48 (0.41–0.56)	<0.01	0.51 (0.43–0.60)	<0.01
Inadequate	39	1		1		1	
Average	563	0.60 (0.43–0.83)	<0.01	0.48 (0.35–0.67)	<0.01	0.52 (0.37–0.73)	<0.01
Optimum	145	0.27 (0.19–0.38)	<0.01	0.23 (0.16–0.34)	<0.01	0.26 (0.18–0.38)	<0.01
*P* for trend		<0.01		<0.01		<0.01	
Each point higher CVH score		0.81 (0.78–0.84)	<0.01	0.78 (0.75–0.81)	<0.01	0.80 (0.77–0.83)	<0.01

Model 1 was a single-factor analysis model; model 2 was adjusted for age, gender, alcohol consumption, income, education and history of cardiovascular disease on the basis of model 1; model 3 was further adjusted for heart rate, uric acid, and high-sensitivity CRP on the basis of model 2.

CVH score, Cardiovascular Health Score; HR, hazard ratio; CI, confidence interval.

### Incidence of Myocardial Infarction in Different Groups

The incidence of myocardial infarction in the groups is shown in Table 3. A total of 747 myocardial infarctions occurred during follow-up. The incidence of myocardial infarction in the three groups was 2.42, 1.44, and 0.65 per 1000 person-years, respectively (Table 2). The hazard ratios (95% CI) of stroke for the average and optimum groups, respectively, in comparison with the inadequate group were: model 1, 0.60 (0.43–0.83) and 0.27 (0.19–0.38); model 2, 0.48 (0.35–0.67) and 0.23 (0.16–0.34); model 3, 0.52 (0.37–0.73) and 0.26 (0.18–0.38). Each better health category of CVH score was associated with reduced odds of 49% for myocardial infarction, and each point higher on the CVH score was associated with reduced odds of 20%. The results indicated that a higher CVH score was negatively related to the incidence of myocardial infarction (*P* for trend <0.01).

### Re-Analysis after Each Metric Was Omitted

To estimate each cardiovascular health metric while accounting for the association between CVH score and CVD incidence, we omitted each metric one at a time and re-analyzed these associations. The residual metrics maintained a significant association with CVD incidence (*P*<0.05). The results of this re-analysis are shown in Table 4.

**Table 4:** Hazard Ratios for CVH Score in Different Groups According to the CVH Score at Baseline when One Cardiovascular Health Metric Is Omitted.

Omitted metric	CVD		Stroke		MI	
	HR (95% CI)	*P*	HR (95% CI)	*P*	HR (95% CI)	*P*
BP	0.66 (0.62–0.71)	<0.01	0.70 (0.65–0.76)	<0.01	0.54 (0.46–0.62)	<0.01
FBG	0.58 (0.54–0.63)	<0.01	0.58 (0.54–0.64)	<0.01	0.56 (0.48–0.66)	<0.01
TC	0.55 (0.51–0.60)	<0.01	0.54 (0.50–0.59)	<0.01	0.58 (0.49–0.67)	<0.01
BMI	0.54 (0.51–0.59)	<0.01	0.55 (0.50–0.60)	<0.01	0.49 (0.42–0.57)	<0.01
Smoking	0.54 (0.51–0.59)	<0.01	0.54 (0.50–0.58)	<0.01	0.57 (0.50–0.67)	<0.01
Physical activity	0.54 (0.51–0.58)	<0.01	0.55 (0.51–0.59)	<0.01	0.51 (0.44–0.59)	<0.01
Salt	0.54 (0.51–0.58)	<0.01	0.56 (0.52–0.60)	<0.01	0.50 (0.43–0.57)	<0.01

CVD, cardiovascular disease; MI, myocardial infarction; HR, hazard ratio (after adjustment for age, gender, alcohol consumption, income, education, history of cardiovascular disease, heart rate, uric acid, and high-sensitivity CRP); CI, confidence interval; BP, blood pressure; FBG, fasting blood glucose; TC, total cholesterol; BMI, body mass index.

## Discussion

CVD has long been regarded as the main threat to public health worldwide [1,2]. Utilizing the scoring system (CVH score) devised by Huffman et al [4], extrapolated from the AHA 2010 definition of ideal cardiovascular health [3], we investigated the possible relationship between CVH score and CVD incidence.

Our results showed that a higher CVH score is related to a decrease in CVD incidence. Each better health category of CVH score was associated with reduced odds of 47% for CVD events, 46% for stroke, and 49% for myocardial infarction. Each point higher on the CVH score was associated with reduced odds of 18% for CVD events, 18% for stroke, and 20% for myocardial infarction. These data are consistent with those of a previous study by Kulshreshtha et al., who followed 22,194 subjects for 4.9 years and found that each better health category of the CVH score was associated with a 25% lower risk of stroke, while a 1-point higher score potentially led to an 8% lower risk of stroke [11]. Also in line with our findings, Xanthakis et al. detected that CVD incidence was inversely associated with CVH score in age- and sex-adjusted models, while each point higher on the CVH score was associated with reduced odds of 16% for cardiovascular events [15]. To the best of our knowledge, several previous studies merely adjusted for age and gender and failed to adjust for other potential risk factors such as heart rate, blood uric acid, and high-sensitivity CRP. However, evidence has proved that the aforementioned factors are independent risk factors for CVD events [16–22]. Furthermore, the present study gives full consideration to the “intermediate” and “poor” health levels of individuals, and further demonstrates the importance of achieving and maintaining a better cardiovascular health status. Our findings suggest that each better health category, or even a 1-point increase in CVH score, can result in substantial reductions in CVD risk. This finding sends an encouraging message regarding health promotion, because intermediate status of cardiovascular health behaviors and factors is a much more realistic target than ideal status for many individuals. If some find it difficult to achieve ideal status, they can nonetheless derive health benefits from reaching the intermediate level, and should be advised as such.

Since the mechanisms underlying this favorable inverse association between CVH score and CVD risk remain unclear, we omitted each metric one at a time and re-analyzed these associations to investigate the effect of each metric. The results indicated that each of the seven metrics is indispensable and is capable of mediating the inverse relationship between CVH score and CVD incidence. Among the seven metrics, BP contributed most to total CVD incidence and stroke, followed by FBG. For myocardial infarction, blood lipids contributed most, with smoking second in rank. Apart from the seven metrics, the mechanisms underlying this association relate to other comprehensive effects. Xanthakis et al. investigated 2,680 participants in the Framingham Offspring Study, and reported that an inverse association between CVH score and CVD incidence was partly attributable to favorable impacts on CVD biomarker levels (such as high-sensitivity CRP) and subclinical diseases [15]. In the present study, we detected a similar trend whereby high-sensitivity CRP, heart rate, and uric acid levels decreased gradually in parallel with the increase in CVH score. Furthermore, the hazard ratios further decreased after adjustment for heart rate, blood uric acid, and high-sensitivity CRP, underlining that the inverse association between CVH score and CVD incidence might partly be due to favorable impacts on these measures. However, the exact mechanisms warrant further exploration.

The AHA proposed the goal of reducing the CVD burden by 20% and increasing cardiovascular health by 20% by the year 2020, but achieving this fig seems to be fairly challenging. At present, the global prevalence of ideal cardiovascular health factors and behaviors is far from satisfactory. Studies in Western countries have found that a mere 0–3.3% of the population met all seven ideal metrics and 4.4–12.2% met five to seven metrics [5–7,10,23–25]. In China, an even worse prevalence of ideal metrics was detected. Wu et al. evaluated 1,012,418 urban residents aged 20–65 years and found that only 0.6% of males and 2.6% of females met all seven ideal health metrics, with 39.1% meeting five to seven metrics [26]. Our previous investigation based on the same cohort (i.e., the Kailuan cohort) also suggested a low prevalence of ideal health metrics. Only 0.6% of subjects met all seven metrics and 9.1% met five to seven metrics [8]. Huffman et al. predicted only a 6% relative improvement in cardiovascular health from 2006 to 2020 in North America if the current trend remains unchanged, a fig far short of the AHA goal of reducing the CVD burden by 20% [4]. The situation regarding cardiovascular health status in China is also unsatisfactory. Our current findings that each better health category of the CVH score was associated with reduced odds of 47% for total CVD events and each point higher on the CVH scale correlated with reduced odds of 18% suggest that a population-wide small improvement in cardiovascular health might have a dramatic impact on reducing the disease burden. Therefore, we should attach more importance to primary prevention when promoting improvement in the cardiovascular health of the population.

There are some limitations to our study. First, this research is based on individual residents in a single community, which might not amply represent the wider population. Second, we were unable to remove the probability of underestimating the incidence of CVD because some subjects never go to hospital for treatment even though they may already be ill. Third, we were also unable to remove the effect of improved cardiovascular health metrics during follow-up. Fourth, we assigned the same weight to the seven metrics in statistical analysis, which may have simplified and underestimated the actual association between CVH score and CVD. Last, we did not document detailed information on daily salt intake.

## Conclusions

Our results give full consideration to the “intermediate” and “poor” status of cardiovascular health metrics, and further demonstrate that a higher CVH score is a protective factor for CVD. Each point higher on the CVH score potentially results in a substantial reduction in CVD risk. Therefore, maintaining an adequate cardiovascular health status among the population is appropriate and desirable in the public health domain.
